# AL-YOLOv8: A Small Object Detection Algorithm for Remote Sensing Images Based on an Improved YOLOv8s

**DOI:** 10.3390/s26072016

**Published:** 2026-03-24

**Authors:** Feng Zhang, Chuanzhao Tian, Xuewen Li, Na Yang, Yanting Zhang

**Affiliations:** 1 North China Institute of Aerospace Engineering, College of Remote Sensing and Information Engineering, Langfang 065000, China; zhangfeng202303@163.com (F.Z.); 15192839463@163.com (X.L.); 19935342291@163.com (N.Y.); zhangyanting0309@163.com (Y.Z.); 2 Collaborative Innovation Center of Aerospace Remote Sensing Information Processing and Application of Hebei Province, Langfang 065000, China

**Keywords:** small object detection, remote sensing images, deep learning, feature fusion, attention mechanism

## Abstract

To address false detections in small object detection within remote sensing imagery caused by complex backgrounds and minute target sizes, we propose an enhanced YOLOv8s detection algorithm, named AL-YOLOv8. The detection head is designed based on Adaptive Spatial Feature Fusion (ASFF) to resolve issues where shallow-level detail features of small remote sensing targets are easily disrupted by backgrounds, while deep-level semantic features lack sufficient representation. We embed Large-Kernel Separate Attention (LSKA) in the deep feature layer to expand the receptive field and enhance the response intensity of small target features. Additionally, an IFIoU loss function is introduced by combining the dynamic attention mechanism from FocalerIoU with InnerIoU, mitigating regression bias for small target bounding boxes and improving small target localization accuracy. On the DIOR, RSOD, and NWPU VHR-10 datasets, the AL-YOLOv8 model achieves precision rates of 91.5%, 94.2%, and 91.8%, respectively, with mAP@0.5 scores of 89.8%, 96.9%, and 92.2%. These results demonstrate consistent improvements over YOLOv8s and show that AL-YOLOv8 effectively reduces false detections and enhances detection accuracy for small object detection in remote sensing applications.

## 1. Introduction

With the rapid advancement of remote sensing technology, remote sensing imagery has found extensive applications in land planning [1], urban management [2], disaster assessment [3], and military reconnaissance [4]. High-resolution remote sensing images typically contain numerous small-scale, densely distributed objects such as vehicles, vessels, and building debris [5]. Accurate identification of these small targets is crucial for subsequent intelligent analysis and decision-making.

Early small object detection in remote sensing imagery largely relied on manual feature methods, such as scale-invariant feature transformations, gradient direction histograms, and deformable part models. While effective for specific tasks, these approaches rely heavily on prior knowledge and have difficulty adapting to the complex, variable object shapes and background environments inherent in remote sensing imagery [6].

In recent years, advancements in computational hardware and the rapid development of deep learning technologies have led to the widespread adoption of deep neural network-based object detection methods for visual tasks. Detection algorithms are typically categorized into two types: two-stage and single-stage approaches. A representative two-stage method is Faster R-CNN [7], which uses a region proposal network to generate candidate boxes, followed by classification and bounding box regression. Single-stage methods, represented by YOLO [8] and SSD [9], employ end-to-end fully convolutional architectures for fast and efficient detection. For small object detection in remote sensing, researchers have modified these general-purpose frameworks, achieving significant improvements. Yu et al. proposed CIMB-YOLOv8, a lightweight variant of YOLOv8, to address complex backgrounds and multi-scale targets in small object detection. By adopting a lightweight group-normalized detail enhancement detection (LGDD) structure with shared convolutions, it reduced parameters by 14% compared to YOLOv8n while improving detection accuracy [10]. Nie et al. proposed a lightweight YOLOv8n-based model for small object detection in remote sensing. They incorporated a dedicated small object detection layer within the feature fusion network and introduced HPANet as an alternative to path aggregation networks to enhance detection accuracy for small objects [11]. Hui et al. introduced SEB-YOLO, an improved algorithm based on YOLOv5 for small object detection in remote sensing images. They employed a Spatial Depth Pooling (SPD) layer and non-stride convolutions (SPD-Conv) to preserve global features while reducing feature loss [12]. Zhao et al. proposed G-YOLO, a lightweight infrared object detection model based on YOLOv8 for drone aerial imaging. They enhanced the YOLOv8 backbone using the lightweight GhostBottleneckV2 architecture and replaced standard convolutions with Deep Separable Convolutions (DWConv). This approach significantly reduced model parameters and computational load while maintaining performance, improving accuracy and efficiency for small object detection [13]. Wu et al. proposed CBGS-YOLO, a small object detection network for remote sensing images based on YOLOv9. They optimized the downsampling process using SPD-Conv modules and integrated a convolutional block attention module, enhancing detection accuracy for small objects in complex backgrounds [14]. Chen et al. proposed the multi-source information fusion framework SVIA DF for small vessel identification and anomaly detection, incorporating the YOLOv8x-CA-CFAR sub-framework based on YOLOv8x. They first detect suspicious targets using YOLOv8x to generate image patches, then perform secondary analysis with CA-CFAR, improving recall and F1-score for small vessel detection by 2.9% and 1.13%, respectively [15]. Wang et al. proposed a dual-component neural architecture incorporating Hierarchical Dynamic Refinement Attention (HiDRA) and Dense Connection Cavity Blocks (DCDBlock) to address limited pixel information and background interference in small object detection for remote sensing [16]. Jin et al. introduced the Dynamic Context Branch Attention Network (DCBANet), a novel backbone architecture for small object detection in remote sensing images. They designed the Dynamic Context Scale Awareness (DCSA) block, which dynamically weights relevant contextual information through a multi-branch architecture and Context-Adaptive Selection Module (CASM) [17]. In addition, the latest dataset construction and benchmarking efforts in the field of autonomous driving provide valuable insights for solving small object detection in complex scenarios. For example, Zunair et al. proposed the RSUD20K dataset, which focuses on complex road scenes in Bangladesh and covers dense traffic flow, severe occlusions, and adverse conditions such as nighttime and rainy weather. This study not only established a large-scale benchmark comprising 13 object classes but also demonstrated through experiments that existing state-of-the-art detectors still face significant performance challenges when handling such high-density, multi-scale targets accompanied by severe occlusions. While RSUD20K is derived from ground-level imagery, the core challenges it presents—namely, detecting dense small objects under complex background interference—share significant similarities with those in remote sensing scenarios [18].

Although existing methods have achieved significant improvements in feature fusion, attention mechanisms, and loss functions, critical research gaps remain: Most approaches focus on optimizing a single module to address one cause of false positives. For example, some rely only on feature fusion to handle cross-scale information inconsistencies, others focus solely on attention mechanisms to enhance small object features, and still others rely only on loss functions to improve localization. These methods have not yet established a comprehensive, end-to-end collaborative optimization strategy targeting the three main causes of false positives: mismatched cross-scale feature spaces, weak small object features in complex backgrounds, and regression bias in bounding box localization. Consequently, they are unable to more effectively address the issue of false positives in complex contexts. The proposed AL-YOLOv8 model overcomes the limitations of existing single-module improvements by implementing progressive collaborative optimization across the feature, attention, and loss layers. It effectively addresses false detection issues in remote sensing small object detection. Key improvements and contributions are as follows:

(1) Designing an Adaptive Spatial Feature Fusion Detection Head: This introduces ASFF [19] technology to reconstruct the detection head. Through a learnable spatial weight allocation mechanism, it dynamically fuses shallow texture and deep semantic information from feature layers of varying scales. This overcomes the limitations of fixed-path fusion in FPN-PAN, enhancing feature discrimination between small targets and complex backgrounds.
(2) Embedding the LSKA [20] Module: Addressing the mismatch between small target receptive fields and object sizes in remote sensing, an LSKA module is embedded before the deep feature output layer. Large-kernel separable convolutions capture long-range dependencies of small targets, while the attention mechanism focuses on sparsely distributed small target regions. This expands the receptive field while controlling computational overhead, strengthening small target feature response.
(3) Proposing the novel bounding box regression loss function IFIoU, to address the insufficient sensitivity of traditional IoU-based losses toward small object bounding boxes. This loss function integrates the central region alignment properties of InnerIoU [21] with the dynamic weighting mechanism of FocalerIoU [22]. By differentially weighting matching errors for small object bounding boxes, it improves localization accuracy for small objects in scenarios with low overlap and proximity to the center, mitigating regression bias issues.
(4) To validate the method’s effectiveness and generalization, this paper utilizes the DIOR [23] dataset covering multi-class complex backgrounds, the RSOD [24] dataset featuring high similarity between small targets and backgrounds, and the NWPU VHR-10 [25] dataset with sparse small target distribution and uneven illumination. Comparative experiments include evaluations against other mainstream models, positional analysis of attention mechanisms, loss function comparisons, ablation studies of individual modules, and detection result comparisons. Experiments demonstrate that the AL-YOLOv8 model achieves stable performance improvements in remote sensing small object detection while effectively reducing false detection rates.

This paper is structured as follows: Section 2 describes the dataset and the AL-YOLOv8 model; Section 3 introduces the experimental environment and evaluation metrics. Section 4 quantitatively and qualitatively analyzes the model’s performance through comparative experiments, ablation studies, and visualization results; Section 5 summarizes the research findings and outlines future directions.

## 2. Materials and Methods

### 2.1. Datasets

This study selected three remote sensing object detection datasets—DIOR, RSOD, and NWPU VHR-10—for experimentation, with a focus on small object detection performance across each dataset. Combining these datasets avoids the scene limitations inherent in a single dataset, ensuring the objectivity of the experimental results and the practical applicability of the models. The definition of small objects in this paper adopts the criteria proposed by Krishna and Jawahar [26], where the bounding box of a small object should cover less than 1% of the original image. In this study, the image dimensions of all datasets were standardized to 640 × 640, fully adhering to the official YOLO default preprocessing logic. No cropping operations were performed at any stage, and the experimental results clearly demonstrate that this strategy of standardizing image dimensions effectively preserves sufficient key pixel information.

**Figure 1:**
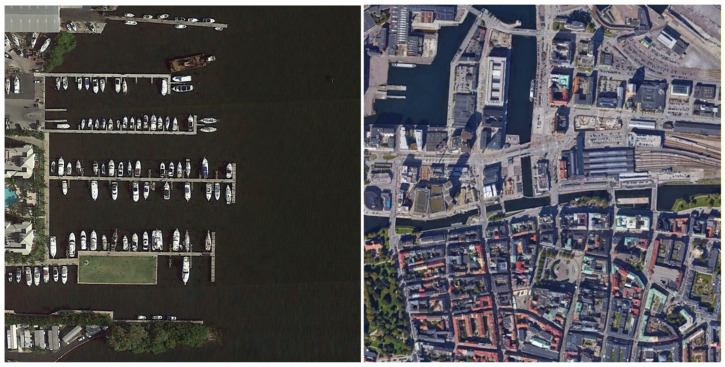
DIOR Dataset Training Sample Display.

DIOR Dataset Developed and released by Wuhan University, this dataset contains 20 object categories (golf field, Expressway toll station, vehicle, train station, chimney, ship, storage tank, harbor, airplane, ground track field, tennis court, dam, stadium, Expressway Service area, airport, baseball field, bridge, windmill, overpass, basketball court), totaling 23,463 remote sensing images. Image sources encompass multiple platforms, including Google Earth and GF-1, with spatial resolutions ranging from 0.5 to 30 m. As a large-scale, multi-category, multi-scenario dataset, DIOR features complex background interference, multi-scale target distribution (ranging from windmills as small as 20 × 20 pixels to bridges as large as 200 × 200 pixels), and dense distributions of small objects. A single image contains multi-class, multi-scale, densely distributed targets, rigorously testing a model’s small object detection capability in such scenarios while preventing models from being effective only in single, simple scenarios. Selected training samples are shown in Figure 1.

**Figure 2:**
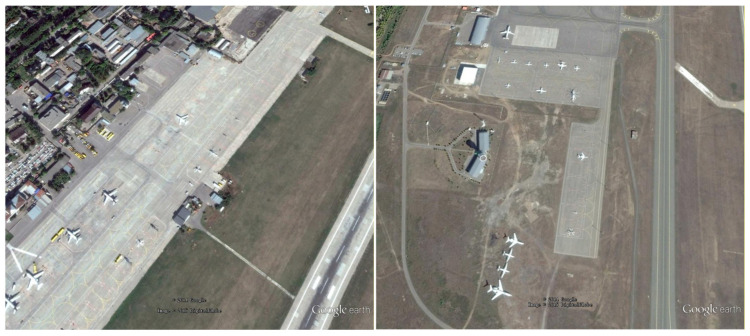
RSOD Dataset Training Sample Display.

The RSOD dataset comprises 976 remote sensing images covering four typical categories: aircraft, oil tanks, overpasses, and playgrounds. The RSOD dataset features single-category objects, but the small targets are scattered and closely resemble the background color. This dataset specifically evaluates a model’s detection accuracy for specific small objects, representing a classic challenge in remote sensing small object detection. It precisely tests a model’s ability to capture small object features. Selected training samples are shown in Figure 2.

**Figure 3:**
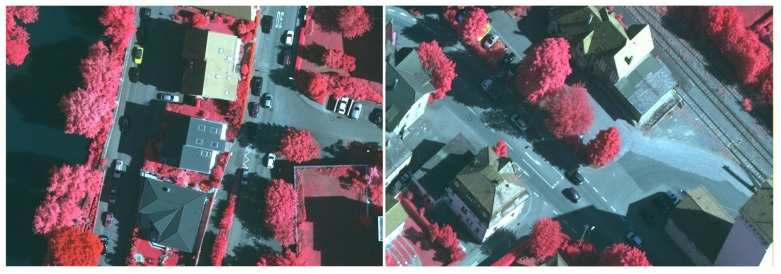
NWPU VHR-10 Dataset Training Sample Display.

The NWPU VHR-10 dataset, constructed and released by Northwestern Polytechnical University, contains 10 common object categories (airplane, baseball diamond, tennis court, basketball court, ground track field, vehicle, storage tank, bridge, harbor, ship). It comprises 650 remote sensing images with spatial resolutions ranging from 0.5 to 2 m. The NWPU VHR-10 dataset features small, scattered objects, uneven illumination, and occlusion, enabling targeted validation of a model’s ability to capture sparse small objects, maintain feature robustness under complex lighting conditions, and recognize objects in occluded scenarios. Selected training samples are shown in Figure 3.

**Figure 4:**
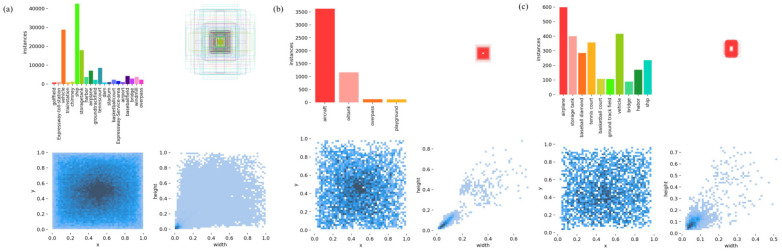
Target distribution. (**a**–**c**) show the DIOR, RSOD, and NWPU VHR-10 datasets, respectively, displaying the number of targets per category, bounding box distribution, center point distribution, and target size distribution.

All three datasets were split into training, validation, and test sets in a 7:2:1 ratio. Figure 4 visualizes the distribution of object categories across the DIOR, RSOD, and NWPU VHR-10 datasets.

To clarify the small target categories prioritized in the experiment, Table 1, Table 2, and Table 3, respectively, summarize the distribution and proportion of small targets within the DIOR, RSOD, and NWPU VHR-10 datasets:

The dataset used in this paper contains small objects accounting for 74% to 100% of the total, exhibiting varying degrees of scale distribution imbalance. To address the issue of uneven distribution between small and medium/large objects in the dataset, the proposed model possesses inherent adaptability through its structural design. The multi-scale feature fusion architecture simultaneously enhances the feature representation of small objects and the semantic information of large objects, preventing the model from favoring one object category over another in scale-imbalanced data. Large-kernel convolutions and attention mechanisms enhance feature responses to small-scale candidate regions, maintaining stable detection performance even in scenarios with low small object ratios. This prevents the model from neglecting small objects due to the dominance of large objects. The optimized loss function further balances regression weights across different-scale objects, ensuring the model retains strong generalization and robustness in imbalanced datasets where small objects are not predominant.

### 2.2. YOLOv8

YOLOv8 [27] is an advanced object detection framework that combines high inference speed, high detection accuracy, and a compact architecture. Its overall design comprises three major components: Backbone, Neck, and Head. The Backbone extracts low-level features from input images through multiple convolutional modules and the newly introduced Cross Stage Partial with 2 Fusion (C2f) module. It incorporates Spatial Pyramid Pooling-Fast (SPPF) at the end to expand the receptive field. This architecture builds upon CSPDarknet, further enhancing feature fusion capabilities and overall detection efficiency. The Neck, positioned between the Backbone and Head, integrates Feature Pyramid Network (FPN) and Path Aggregation Network (PAN) structures to achieve effective fusion of multi-scale features, thereby significantly enhancing the model’s recognition performance for objects of various scales. The Head comprises three decoupled modules, each independently performing object classification and bounding box regression tasks to enable more precise multi-task learning [28,29]. Unlike traditional anchor-based methods, YOLOv8 employs an Anchor-Free mechanism. This eliminates the need for predefined anchor boxes, directly predicting object center points and related parameters. This approach significantly boosts inference speed while maintaining detection accuracy. To adapt to diverse hardware environments and application scenarios, YOLOv8 offers five progressively scaled model variants: v8n, v8s, v8m, v8l, and v8x [30]. Key differences between versions lie in the number of feature extraction modules and convolutional kernel configurations. While larger models exhibit stronger feature representation capabilities, computational complexity increases substantially. YOLOv8s strikes a balance between detection accuracy, parameter count, and computational load, making it suitable for lightweight deployment in small object detection for remote sensing. This paper therefore introduces targeted enhancements tailored to these requirements.

### 2.3. AL-YOLOv8

**Figure 5:**
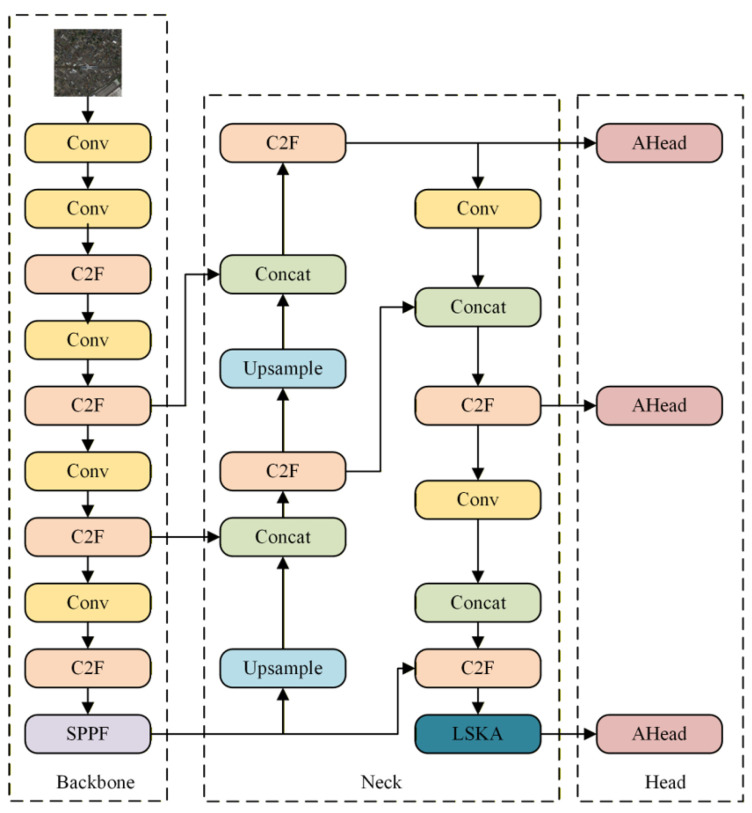
AL-YOLOv8 Model Architecture.

This study conducted the following optimizations based on the YOLOv8s framework: an adaptive spatial feature fusion mechanism was integrated into the detection head, combining the boundary-fitting advantages of InnerIoU with the focus-on-difficult-samples characteristics of FocalerIoU to construct a new hybrid localization loss function, IFIoU. ASFF enables dynamic multi-scale feature fusion, providing a rich feature foundation for LSKA; LSKA enhances global feature responses for small objects, improving feature discrimination; the IFIoU loss function precisely optimizes bounding box localization. These three components form a synergistic enhancement, progressing from features to localization. Figure 5 illustrates the improved network architecture. This model’s feature extraction follows a differentiated expression mechanism from shallow to deep layers: shallow detail features primarily originate from the first two C2f modules of the backbone network and lower convolutional layers, responsible for capturing fine-grained structural information such as edges, textures, and contours of small remote sensing targets; deep semantic features are provided by the C2f modules in the last two layers of the backbone network and the SPPF output layer, focusing on high-level semantic information such as target categories and context. The model achieves complementary integration of detail and semantic information by weighting and fusing these shallow-level detail features with deep-level semantic features through the multi-scale feature fusion module at the Neck end.

#### 2.3.1. Improvements to the Detection Head

In remote sensing small object detection, target sizes predominantly fall within the 10–50-pixel range amid strong background interference. The FPN-PAN feature fusion mechanism employed by YOLOv8 exhibits limitations, as it relies on fixed top-down or bottom-up information propagation paths coupled with scale alignment strategies involving upsampling and concatenation. This approach fails to dynamically allocate feature weights based on the spatial distribution variations inherent to remote sensing of small objects. When handling dense small targets, this mechanism is prone to issues such as shallow-level detail features being drowned out by the background, loss of deep-level semantic features, feature redundancy, and missing small target information, ultimately compromising detection accuracy. To address this shortcoming, this paper introduces an ASFF detection head. ASFF employs a spatially learnable weight allocation mechanism to dynamically select optimal features for each pixel location based on semantic requirements. This compensates for the limitations of traditional fixed-weight fusion and better adapts to the scattered spatial distribution and sparse features characteristic of small remote sensing targets.

**Figure 6:**
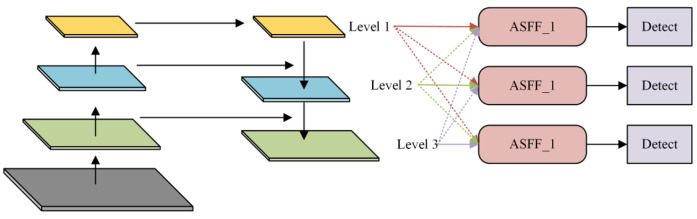
ASFF Module Structure.

The specific improvement approach is as follows: The three-scale features (shallow details, mid-level balance, deep semantics) output by the YOLOv8 backbone network are first unified in channel count via 1 × 1 convolutions. They are then aligned to the spatial dimensions of the current detection layer through upsampling and downsampling. The aligned features are then fed into the ASFF module, which applies learned spatial weight maps to fuse multi-scale features with dynamic adjustment. Unlike conventional concatenation, ASFF achieves adaptive allocation of cross-scale information flow in the spatial dimension. For pixels containing small targets, the network automatically increases the weight of shallow features to preserve details. For regions with complex backgrounds, it boosts the weight of deep features to enhance semantic discrimination, thereby improving recognition of blurred small targets and those near boundaries. The fused feature map is fed into a decoupled detection head for classification and regression tasks: the decoupling head separates features for classification and regression branches, preventing interference between small object classification features and localization features, thereby further enhancing context modeling and task decoupling capabilities. The ASFF mechanism is illustrated in Figure 6.

For layer l, the feature fusion process of ASFF can be represented as follows:

(1) $$y_{ij}^{l}=\alpha_{ij}^{l}*x_{ij}^{1\to l}+\beta_{ij}^{l}*x_{ij}^{2\to l}+\gamma_{ij}^{l}*x_{ij}^{3\to l}$$

In this context, $y_{ij}^{l}$ represents the feature vector at position (i,j) in the fused feature map of the l layer; $x_{ij}^{1\to l}$, $x_{ij}^{2\to l}$, and $x_{ij}^{3\to l}$ represent the feature vectors at position (i,j) in the three input feature maps. The weights $\alpha_{ij}^{l}$, $\beta_{ij}^{l}$, and $\gamma_{ij}^{l}$ denote the spatial weight at this location, representing the contribution of features at different scales to the current position. These weights are learned through the convolutional network and normalized using the Softmax function, satisfying the following:

(2) $$\alpha_{ij}^{l}+\beta_{ij}^{l}+\gamma_{ij}^{l}=1$$

The normalized form is as follows:

(3) $$\alpha_{ij}^{l}=\frac{e^{\lambda_{ij}^{l}}}{e^{\lambda_{ij}^{l}}+e^{\mu_{ij}^{l}}+e^{\nu_{ij}^{l}}}$$

$\lambda_{ij}^{l}$, $\mu_{ij}^{l}$, and $\nu_{ij}^{l}$ is the learnable weight parameter for the output of a 1 × 1 convolution.

This design employs spatially adaptive weight adjustment, enabling the network to dynamically enhance key features based on the spatial distribution of small remote sensing targets, thereby improving robustness toward small targets at varying scales.

#### 2.3.2. LSKA Module

In remote sensing small object detection, background textures easily obscure target features, making it challenging to sufficiently enhance small object feature responses through feature fusion alone. To address this, this paper incorporates a LSKA module before the detector head to expand the effective receptive field while preserving small object detail features.

**Figure 7:**
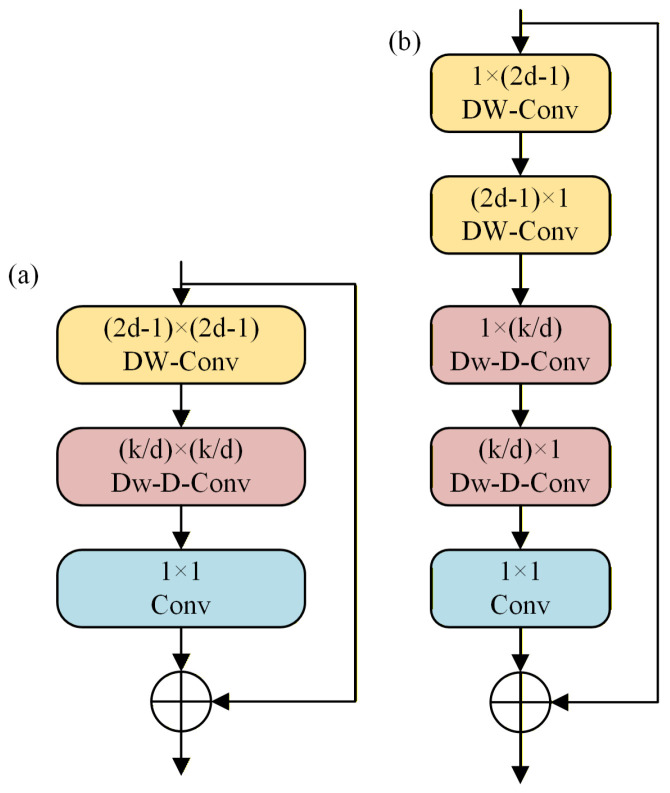
(**a**) LKA module structure; (**b**) LSKA module structure.

LSKA constructs an attention structure based on large-scale separable convolutional kernels, optimized to address the adaptation deficiencies of traditional LKA in small target scenarios. Traditional LKA modules typically extract local spatial information via (2d − 1) × (2d − 1) depth convolutions, followed by capturing global information through k/d × k/d dilation convolutions. While this structure expands the receptive field, its reliance on large-sized 2D convolutional kernels tends to over-capture background texture information in remote sensing images, obscuring the weak features of small targets. To address this, LSKA decomposes the large 2D kernel convolution in LKA into serial one-dimensional separable convolutions along horizontal and vertical directions. Replacing parallel 2D operations with serial 1D computations enables more precise capture of fine-grained features like edges and textures in small remote sensing targets. This design enhances feature discrimination between targets and backgrounds by mitigating background texture interference while reducing computational complexity from the original 2D convolution and expanding the effective receptive field to cover the spatial distribution of small targets while adapting to YOLOv8s’ lightweight architecture. Furthermore, the spatial information accumulation from serial 1D convolutions effectively captures long-range contextual information of small targets, compensating for their inherent feature sparsity. The LSKA and LKA structures are illustrated in Figure 7. Compared to the LKA module, LSKA achieves a structural balance between receptive field expansion and detail preservation, making it more suitable for detecting small targets in remote sensing. This further enhances the model’s sensitivity and expressive capability regarding small target features.

#### 2.3.3. Loss Function Improvement

Small target bounding boxes in remote sensing have minimal dimensions, and positioning errors significantly impact detection accuracy. Simultaneously, sample imbalance arises where easily classifiable samples dominate while challenging small targets are overlooked. To address this, this paper proposes an improved positioning loss function, IFIoU. By integrating InnerIoU’s central region focus mechanism with FocalerIoU’s sample weighting strategy, it simultaneously enhances small target localization accuracy and increases the model’s attention to challenging samples.

**Figure 8:**
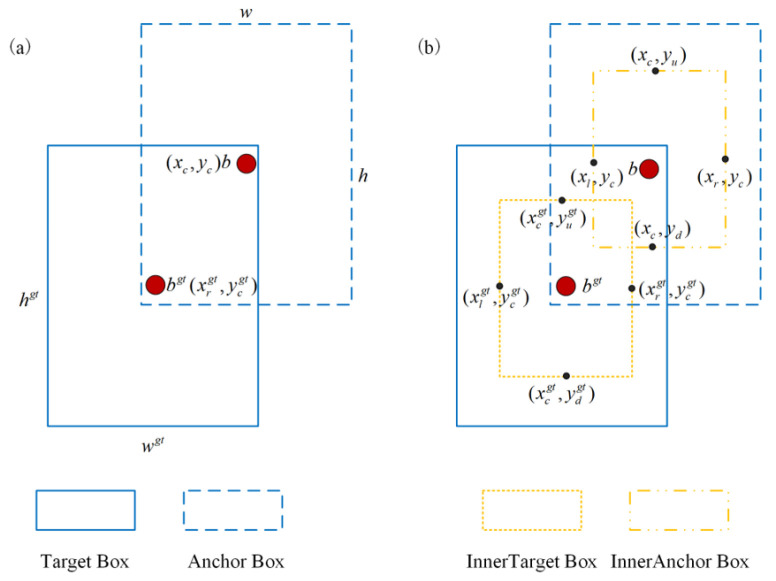
A schematic of the sample matching mechanism based on inner and outer boxes. (**a**) The standard IoU matching process. (**b**) The structure with the introduction of auxiliary boxes.

The core design of IFIoU comprises two parts. First, it introduces InnerIoU’s inner scaling auxiliary mechanism. Within the anchor box and target box, it generates more compact auxiliary anchor boxes (InnerAnchor Box) and auxiliary target boxes (InnerTarget Box) centered on the box center and scaled by a ratio factor. This focuses on the overlap degree in the core region. This mechanism better accommodates the characteristics of small remote sensing targets, where the central region holds more critical features. It enhances alignment accuracy between prediction boxes and target boxes in the core area, preventing localization errors caused by low overall overlap of small target boxes. As shown in Figure 8, auxiliary boxes are generated centered on the intersection of the ground-truth and predicted boxes, with scaling controlled by the parameter ratio (where ratio ∈ (0,1); smaller values concentrate the focus area). Their boundary coordinates are computed by proportionally scaling the original box’s width and height. InnerIoU is then calculated based on these auxiliary box coordinates to quantify overlap in the central region. Second, we incorporate the Focal concept to establish a FocalerIoU weighting mechanism, transforming the InnerIoU value through a piecewise linear mapping function: When InnerIoU falls within the preset range [d, u], it undergoes stretching mapping to amplify the loss weight for samples with ambiguous localization. Samples with excessively low or high IoU are assigned fixed weights of 0 or 1, respectively. This approach adaptively enhances attention to small, hard-to-classify targets while reducing the loss contribution from easily classified samples, mitigating the impact of sample imbalance on detection performance. In this context, d and u are used to define the mapping interval for InnerIoU, playing a crucial role in weight allocation for challenging samples. Based on the localization characteristics of small remote sensing targets, this study ultimately determined d = 0.00 and u = 0.95 as the optimal threshold combination. For adaptability analysis of small remote sensing targets, setting d to 0.00 fully covers all low-overlap samples, preventing insufficient weight allocation for such samples due to excessively high thresholds. Setting u to 0.95 maximizes coverage of normal-overlap samples while avoiding excessive weight stretching for high-overlap easy samples, balancing training efficiency and accuracy.

The center coordinates of the ground-truth box are ($x_{c}^{gt}$, $y_{c}^{gt}$) with width $w^{gt}$ and height $h^{gt}$. The four boundary coordinates of the auxiliary box InnerTarget are calculated as follows:

(4) $$x_{l}^{gt}=x_{c}^{gt}-\frac{w^{gt}\cdot ratio}{2},\;x_{r}^{gt}=x_{c}^{gt}+\frac{w^{gt}\cdot ratio}{2}$$

(5) $$y_{u}^{gt}=y_{c}^{gt}-\frac{h^{gt}\cdot ratio}{2},\;y_{d}^{gt}=y_{c}^{gt}+\frac{h^{gt}\cdot ratio}{2}$$

The center coordinates of the prediction box are ($x_{c}$, $y_{c}$) with width and height to be specified. The coordinates of the generated auxiliary box InnerAnchor are as follows:

(6) $$x_{l}=x_{c}-\frac{w\cdot ratio}{2},\;x_{r}=x_{c}+\frac{w\cdot ratio}{2}$$

(7) $$y_{u}=y_{c}-\frac{h\cdot ratio}{2},\;y_{d}=y_{c}+\frac{h\cdot ratio}{2}$$

Herein, the parameter ratio ∈ (0,1) is used to control the scaling degree of the auxiliary box; the smaller its value, the smaller the focused region will be.

Based on the region defined by the auxiliary box, the calculation formula of InnerIoU is given as follows:

(8) $${IoU}_{inner}=\frac{inter}{union}$$

Herein, the intersection area (inter) and the union area (union) are calculated as follows, respectively:

(9) $$inter=(\min(x_{r}^{gt},x_{r})-\max(x_{l}^{gt},x_{l}))\cdot (\min(y_{d}^{gt},y_{d})-\max(y_{u}^{gt},y_{u}))$$

(10) $$union=w^{gt}\cdot h^{gt}\cdot {ratio}^{2}+w\cdot h\cdot {ratio}^{2}-inter$$

Let $x_{r}^{gt}$, $x_{l}^{gt}$, $y_{d}^{gt}$, and $y_{u}^{gt}$ denote the boundary coordinates of the auxiliary box (InnerTarget) for the ground-truth box; $x_{r}$, $x_{l}$, $y_{d}$, and $y_{u}$ denote the boundary coordinates of the auxiliary box (InnerAnchor) for the prediction box; $w^{gt}$, $h^{gt}$, denote the width and height of the ground-truth box; and w, h, denote the width and height of the prediction box. To enhance the model’s focus on hard samples, the FocalerIoU linear piecewise mapping function is introduced to convert InnerIoU values into final IoU values:

(11) $$IoU=\left\{\begin{array}{cc} 0, & {IoU}_{inner}<d\\ \frac{{IoU}_{inner}-d}{u-d}, & d\leq {IoU}_{inner}\leq u\\ 1, & {IoU}_{inner}>u \end{array}\right.$$

Herein, d, u denote the upper and lower thresholds of the mapping interval, respectively, whose purpose is to stretch the intermediate value region, thereby focusing on target boxes with ambiguous localization.

Combined with the aforementioned mapping, the final regression loss function is given as follows:

(12) $$L_{IFIoU}=1-IoU$$

Although IFIoU formally combines InnerIoU and FocalerIoU, its conceptual originality lies in its collaborative integration mechanism designed to address the unique challenges of small target detection in remote sensing. Unlike simple linear aggregation, IFIoU aims to resolve the dual dilemma in small target detection: namely, spatial sensitivity (where even slight shifts in small bounding boxes can cause a sharp drop in IoU) and sample imbalance (where hard-to-locate small targets are easily overwhelmed by a large number of simple background samples).

Specifically, the InnerIoU component provides strict spatial constraints by focusing on the core region of the bounding box, forcing the model to learn more precise center alignment and thereby alleviating the ambiguity in small target localization; meanwhile, the FocalerIoU component introduces dynamic, difficulty-aware weights that adaptively amplify the gradient contributions of difficult samples with low overlap that are hard to regress.

This design creates a complementary feedback loop: it not only tightens the spatial constraints on the critical core regions of small targets but also ensures that samples difficult to localize due to complex backgrounds or occlusions receive sufficient attention during training. Consequently, IFIoU goes beyond incremental improvements, providing a unified regression framework capable of simultaneously optimizing both the localization accuracy of sparse small targets and the stability of convergence.

## 3. Experimental Setup and Evaluation Metrics

### 3.1. Model Training Device and Parameter Setup

The hardware configuration used for the experiment consists of an Intel^®^ Core™ i7-14650HX (Intel Corporation, Santa Clara, CA, USA) processor with a base frequency of 2.20 GHz, 32 GB of memory, and an NVIDIA RTX 4060 (NVIDIA Corporation, Santa Clara, CA, USA) graphics card with 8 GB of RAM.

The software environment includes the Windows 11 operating system, the PyTorch 1.12.1 deep learning framework, conda version 25.5.1, and Python 3.9.20. Experimental parameters are set as follows: image_size = 640, training epochs = 200, batch size = 8, and learning rate = 0.01. Detailed training parameters are shown in Table 4.

### 3.2. Evaluation Metrics

The experiment employs four widely used evaluation metrics: Precision, Recall, Average Precision (AP), and Mean Average Precision (mAP). Precision reflects the proportion of true positives among all predicted positive samples; Recall measures the proportion of true positives successfully identified. AP represents the area under the P-R curve for a specific category, reflecting detection performance for that target class. mAP is the average of AP across all categories, serving as a metric for overall performance. The commonly used mAP@0.5 refers to the average AP calculated at an IoU threshold of 0.5, a key indicator for evaluating localization capability in the object detection domain. This paper also reports mAP@[0.5:0.95], calculated by averaging AP across IoU thresholds from 0.5 to 0.95 (in 0.05 increments). This metric is more rigorous and comprehensive, enabling a more precise evaluation of model performance across different levels of localization accuracy. The evaluation metrics are defined as follows:

(13) $$P=\frac{TP}{TP+FP},\;R=\frac{TP}{TP+FN},\;AP=\int_{0}^{1}P(r)dr,\;mAP=\frac{1}{N}\sum_{i=1}^{N}APi$$

True Positives (TPs) represent the number of samples predicted as positive by the model that are actually positive; False Positives (FPs) represent the number of samples predicted as positive by the model that are actually negative; False Negatives (FNs) represent the number of samples predicted as negative by the model that are actually positive; N denotes the total number of samples in the category.

## 4. Results

### 4.1. Changes in Various Metrics Throughout the Training Process

**Figure 9:**
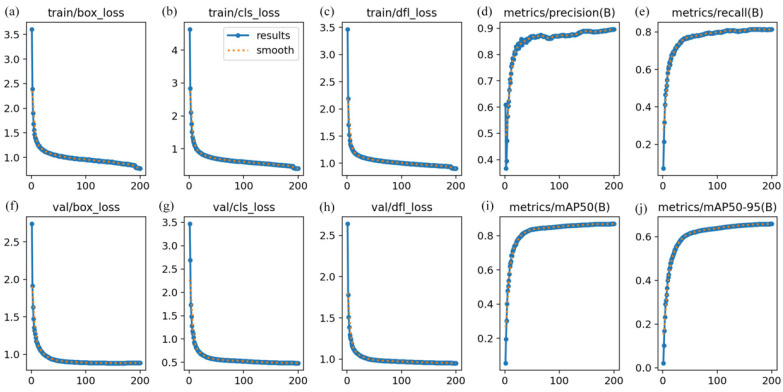
Key metric change curves during model training. (**a**–**c**) Changes in box, cls, and dfl losses on the training set; (**d**,**e**) precision and recall curves; (**f**–**h**) box, cls, and dfl losses on the validation set; (**i**,**j**) changes in mAP@0.5 and mAP@0.5:0.95.

To validate the convergence and performance stability of the improved model in remote sensing small object detection tasks, Figure 9 illustrates the loss function evolution and key performance metric curves of AL-YOLOv8 during 200 training epochs on the DIOR dataset. During both training and validation phases, localization loss (box_loss), classification loss (cls_loss), and distribution fitting loss (df1_loss) all exhibit a sustained downward trend, indicating progressive optimization in the model’s localization accuracy, category discrimination, and distribution feature fitting capabilities for small remote sensing targets. Notably, box_loss and df1_loss decrease significantly within the first 50 epochs before stabilizing. This pattern reflects both the rapid convergence of the regression process and aligns with the training characteristics of small remote sensing target localization, where initial losses decrease rapidly due to high difficulty, followed by a need for fine-tuning in later stages.

Performance metrics show steady upward trends in Precision and mAP@0.5 curves: improved Precision indicates reduced false positives (background misclassified as targets), while continuous mAP@0.5 optimization demonstrates the model’s balanced performance between detection accuracy and coverage. This result further validates the proposed method’s robustness and generalization capability in multi-class small remote sensing object detection tasks. It also demonstrates that the improved algorithm exhibits stable training and clear performance gains on the DIOR dataset, laying the foundation for subsequent validation of the method’s effectiveness on diverse remote sensing datasets such as RSOD and NWPU VHR-10.

### 4.2. Comparative Experiment

#### 4.2.1. Comparison with Other Algorithms

**Figure 10:**
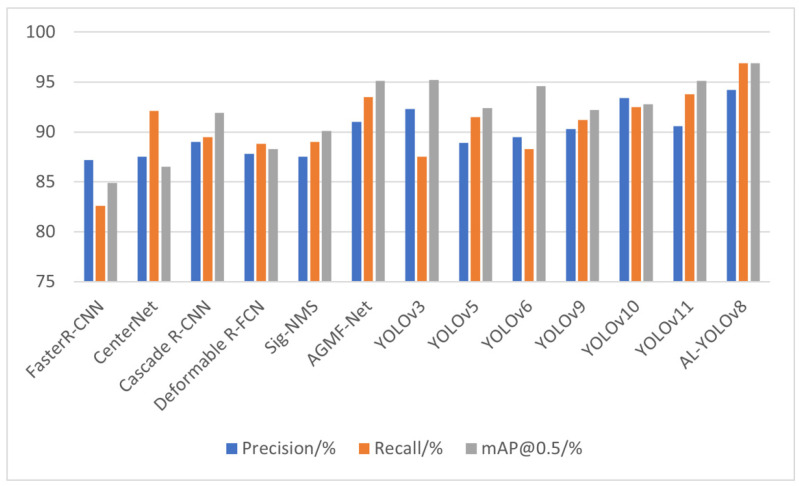
Performance comparison of various models across Precision, Recall, and mAP@0.5 metrics.

To validate the effectiveness of the improved algorithm, it was compared with Faster R-CNN, CenterNet [31], YOLOv3, YOLOv5, YOLOv6, YOLOv9, YOLOv10, and YOLOv11 on the RSOD dataset. Additionally, to comprehensively evaluate the competitiveness of our method in small object detection scenarios for remote sensing, we also introduced representative models for small object detection in remote sensing imagery for comparison, including Cascade R-CNN [32], Deformable R-FCN [33], Sig-NMS [34], and AGMF-Net [35]. To ensure a fair comparison, all of the above comparison models used the same training framework and hyperparameter settings as AL-YOLO. The results are shown in Figure 10 and the corresponding Table 5.

In terms of precision, the improved algorithm achieved an accuracy of 94.2%, demonstrating superior performance in reducing false positives compared to other models. Regarding Recall, our method achieves 93.8%, significantly surpassing the other comparison methods, and demonstrating a clear advantage in reducing missed detections. Regarding the mAP metric, the proposed method also achieved the highest value of 96.9%, representing a 1.8 percentage point improvement over YOLOv11. This fully demonstrates the model’s enhanced overall detection performance. The improved algorithm outperforms multiple existing mainstream object detection algorithms in terms of P, R, and mAP@0.5, validating its superiority in target localization accuracy and detection robustness.

#### 4.2.2. LSKA Module Embedding Position Comparative Analysis

To determine the optimal embedding position of the LSKA module within the YOLOv8s network and investigate the impact of feature layer embedding locations on small object detection performance in remote sensing, this study conducted comparative experiments by selecting four typical candidate positions within YOLOv8’s feature fusion layer: before P4 upsampling, after P3 feature layer, after P4 downsampling, and after P5 feature layer. All experiments were conducted on the RSOD dataset, maintaining consistent parameters while varying only the LSKA module’s embedding position. Quantitative comparisons of precision, recall, and mAP@0.5 metrics across different positions identified the optimal embedding strategy. Experimental results are presented in Table 6.

When the LSKA module is embedded in the P3 feature fusion layer, the model’s metrics are at their lowest. This is because P3 is a shallow feature layer in YOLOv8, primarily capturing fine-grained details such as object edges and textures, lacking sufficient semantic information support. The large convolutional receptive field of LSKA cannot function effectively here, making it difficult to extract global features of small objects efficiently.

When embedded into both the pre-upsampling and post-downsampling P4 feature layers, model performance improved. As an intermediate feature layer, P4 balances detailed and semantic features, better accommodating LSKA’s feature enhancement mechanism. However, it still falls short in extracting deep semantic features of small remote sensing targets. When the LSKA module is embedded into the deep feature layer P5, the model achieves optimal overall detection performance. This layer, corresponding to YOLOv8’s deep feature layer, contains rich semantic information and global features. Embedding LSKA here allows its large receptive field to fully capture long-range dependencies of small remote sensing targets, effectively compensating for the lack of fine-grained details in deep feature layers while enhancing the representation capability of small targets against complex backgrounds.

**Figure 11:**
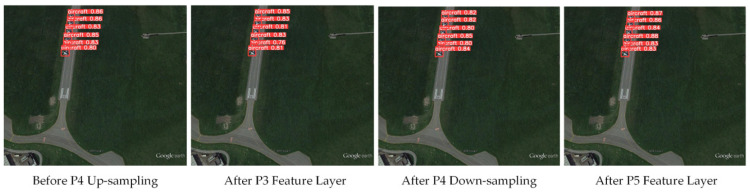
Effects of Different Feature Layers and Up/Downsampling Operations on Small Object Detection.

To more intuitively validate the impact of embedding the LSKA module at different feature layers on small object detection performance and further corroborate the analysis results, we supplemented the visualization of small object detection outcomes at various embedding positions. We compared the detection confidence and bounding box localization effectiveness for aircraft targets across different embedding locations. As shown in Figure 11, when the LSKA module is embedded after the P3 feature layer or before the P4 upsampling stage, some aircraft targets exhibit relatively low detection confidence and exhibit slight misalignment in bounding box localization. Conversely, embedding the LSKA module after the deep P5 feature layer significantly enhances detection confidence for all aircraft targets and improves the alignment accuracy between bounding boxes and ground-truth objects. This intuitively demonstrates that embedding the LSKA module in the deep feature layer P5 can more effectively enhance small target feature responses and optimize localization accuracy.

#### 4.2.3. Loss Function Comparison

**Figure 12:**
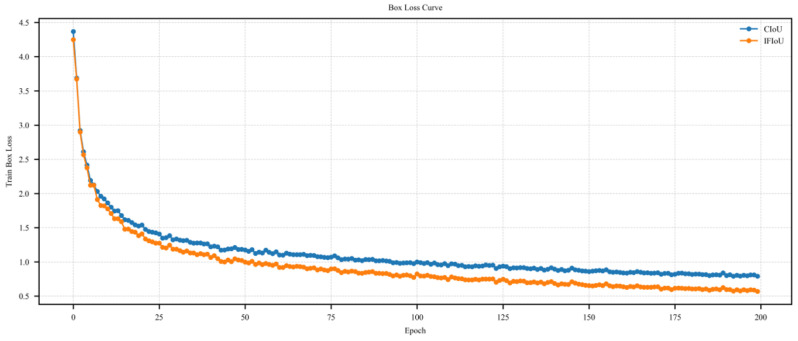
Box Loss Convergence Curve Comparison.

As shown in Figure 12, on the DIOR dataset, the box loss convergence curves during the training phase for the CIoU loss used in the original YOLOv8 and the IFIoU loss proposed in this paper. Compared to CIoU, IFIoU converges faster during the initial phase and maintains lower box loss values throughout the entire training process, indicating superior fitting capability for bounding box regression tasks. In the mid-to-late stages, IFIoU exhibits smoother loss reduction, demonstrating enhanced stability and optimization effects, thereby further improving detection box localization accuracy.

The ratio parameter in IFiOU controls the scaling ratio of the core region auxiliary box, directly affecting the model’s localization accuracy for the core regions of small targets. This study employs grid search to determine the optimal value of ratio within the range of 0.1 to 0.9 (step size 0.1). Using the RSOD dataset’s mAP@0.5 as the evaluation metric, we test how different ratio values affect the model’s overall detection performance and its detection of small objects at various scales. The results are shown in Table 7. The grid search results indicate that when ratio = 0.7, the model achieves optimal detection performance both overall and for small targets at different scales: an excessively small ratio causes the core region to focus too narrowly, losing the edge features of small remote sensing targets; an excessively large ratio causes the core region to expand excessively. Therefore, this study ultimately sets the ratio parameter to 0.7.

To further validate the positioning accuracy advantages of the IFIoU loss function in remote sensing object detection, we conducted comparative experiments on the RSOD dataset using both CIoU and IFIoU as bounding box regression loss functions. The experimental results are shown in Table 8. For the aircraft category, the model using IFIoU achieves an mAP@[0.5:0.95] reached 60.8%, representing a 0.9 percentage point improvement over CIoU. In the full-category evaluation, IFIOU elevated mAP@[0.5:0.95] from 65.1% to 66.1%, achieving an overall 1.0 percentage point gain. The results demonstrate that IFIoU effectively enhances the model’s localization accuracy for small remote sensing targets by optimizing the constraint mechanism for bounding box regression. It maintains stable performance gains even under stricter IoU threshold evaluations, further validating its effectiveness in improving the detection and localization accuracy of small remote sensing targets.

### 4.3. Experimental Results

Experiments were conducted on three representative remote sensing datasets: DIOR, RSOD, and NWPU VHR-10. Detailed results are as follows. This study focuses on small object detection in remote sensing imagery. Therefore, the results tables for each dataset only present core categories with a high proportion of small objects that are sensitive to the proposed improvements. Other categories dominated by medium-to-large objects, which are less responsive to the performance gains of our method, are not shown individually due to space constraints.

As shown in Table 9, on the DIOR dataset featuring complex terrain features, the model’s overall accuracy improved from 89.7% to 91.5%, directly reflecting a significant reduction in misclassifying background noise as small targets. Particularly for small target categories like vehicles—which feature sparse characteristics and are easily confused with road textures—the accuracy improved from 87.4% to 89.2%, representing a 1.8 percentage point increase. This demonstrates that the enhanced feature fusion and attention mechanism effectively strengthened the distinction between small targets and complex backgrounds, substantially reducing background false detection rates.

To evaluate the stability of model performance, we conducted five independent training and testing experiments on the DIOR dataset. The mean, standard deviation, and 95% confidence interval for key metrics across all categories are shown in Table 10. The results indicate that the standard deviation for all metrics across categories is controlled within 0.6%, with reasonable 95% confidence intervals. This demonstrates that the AL-YOLOv8 model exhibits consistent performance and robustness across different object categories, effectively mitigating the randomness inherent in single-run experiments and validating the reliability of the methodology.

To systematically analyze the suppression effect of the proposed method on false detection of small targets in complex backgrounds, we constructed an error matrix based on 100 typical images from the DIOR test set. The distribution of false alarms before and after improvement was statistically analyzed by false alarm type, with the results shown in Table 11. The table clearly demonstrates: false positives of ships in water backgrounds decreased from 12 to 2, representing an 83.33% reduction; false positives of storage tanks in industrial ground backgrounds decreased from 18 to 4, representing a 77.78% reduction; false positives of aircraft in grassland backgrounds decreased from 28 to 5, representing an 82.14% reduction; the number of false alarms for aircraft detected at the edges of airport buildings decreased from 19 to 4, representing a reduction of 78.95%; the number of false alarms for wind turbines detected in farmland backgrounds decreased from 8 to 1, representing a reduction of 87.50%. These results fully demonstrate that the proposed method can significantly reduce false detection rates and enhance the robustness of small object detection in complex remote sensing scenarios.

As shown in Table 12, on the RSOD dataset featuring small targets with highly similar backgrounds, the model’s overall accuracy improved from 91.6% to 94.2%. For small targets like aircraft—which closely resemble airport runways and open ground backgrounds—accuracy increased by 1.3 percentage points. This demonstrates the model’s enhanced capability to accurately identify core features of small targets in highly similar background scenarios, effectively preventing misclassification of background areas as targets.

As shown in Table 13, on the NWPU VHR-10 dataset, the precision rate for the vehicle category increased from 79.1% to 82.5%, while that for the ship category rose from 77.6% to 81.2%. Both categories saw improvements exceeding 3 percentage points, indicating that the enhanced model reduces misclassification issues caused by scattered targets and uneven illumination. Simultaneously, the overall model accuracy rose to 91.8%, further validating that the enhanced models possess stable resistance to false detections.

### 4.4. Ablation Experiment

To validate the independent roles and collaborative effects of each core module within the proposed network, ablation experiments were conducted on the RSOD dataset by progressively introducing the ASFF, LSKA, and IFIoU modules. The results are shown in Table 14.

YOLOv8s, serving as the baseline model in this study, inherently possesses outstanding lightweight and real-time capabilities. With only 11.14 million parameters and 14.33 GFLOPs, it achieves an inference frame rate of 114.1 FPS—significantly above the typical latency threshold for real-time remote sensing detection. This characteristic makes it an ideal choice for resource-constrained scenarios such as drones and edge monitoring terminals. It ensures detection efficiency while providing a robust lightweight foundation for subsequent accuracy improvements.

When introducing the ASFF module alone, the model’s accuracy improved to 93.5% and recall to 92.5%. Concurrently, the model’s parameters increased from the baseline 11.14 million to 16.58 million. The FLOPs increased from 14.33 G to 18.55 G, and the FPS decreased from 114.1 to 87.3, yet it still maintained a frame rate significantly higher than the requirements for real-time remote sensing detection. The ASFF module effectively mitigates the misalignment of cross-scale feature representations for small remote sensing targets through its adaptive spatial weight allocation mechanism. It dynamically adapts to the feature requirements of small targets at different scales, enabling efficient interaction and complementarity between shallow-layer fine-texture features and deep-layer semantic features. This approach enhances the feature distinction between small targets and background noise, reducing misclassification of background areas as targets, while also addressing the weakness of traditional fixed-path fusion in handling sparse small target features. After introducing the LSKA module alone, the model’s mAP@0.5 improved to 95.7%. Meanwhile, the model’s parameter count increased only slightly from 11.14 M to 11.41 M (an increase of approximately 2.4%), FLOPs rose marginally from 14.33 G to 14.43 G (approximately 0.7% increase), while FPS improved from 114.1 to 115.7. This demonstrates the design advantage of the large-core separable attention module: significantly expanding the effective receptive field with negligible additional computational overhead. The large-core architecture of the LSKA module substantially expands the model’s receptive field, enabling precise capture of subtle and sparse feature regions in small remote sensing targets. It enhances feature response intensity in critical target areas while suppressing interference from complex background textures. When introducing the IFIoU hybrid loss function alone, model recall improved to 92.7%. Meanwhile, model parameters, FLOPs, and FPS remained largely consistent with the baseline model. This demonstrates that IFIoU, when optimized as a pure loss function, imposes no additional computational burden. It enhances the regression accuracy of small object bounding boxes solely through loss weighting strategies. IFIoU combines the central region alignment advantage of InnerIoU with the adaptive weighting feature for challenging samples in FocalerIoU. Addressing the pain points of small object bounding box size and positioning error sensitivity in remote sensing, it employs inner scaling to assist the bounding box in focusing on the target’s core region. dynamically reinforcing regression constraints for small target bounding boxes. It also applies differential weighting to losses for hard-to-classify small targets with low overlap or edge occlusion, thereby improving regression accuracy and reducing false detections caused by ambiguous localization.

In the combined module ablation experiment, simultaneously introducing ASFF and LSKA further improved the model’s accuracy to 93.6% and recall to 92.8%. The number of parameters increased to 18.66 million, FLOPs rose to 16.85 billion, and FPS decreased to 90.8, while maintaining real-time detection capability. This demonstrates synergistic enhancement between the two modules: ASFF’s cross-scale feature fusion provides a more comprehensive and high-quality feature foundation for LSKA’s attention enhancement, while LSKA focuses and amplifies key regions within the fused features. This progressive relationship further optimizes small object feature expression quality and strengthens target-background discrimination. When both ASFF and IFIoU are introduced, the model achieves a recall rate of 93.0% and a mAP@0.5 of 96.2%. with parameter counts and FLOPs matching standalone ASFF while reducing FPS to 86. This demonstrates the complementary nature of feature fusion and localization loss optimization: ASFF-enhanced multi-scale features provide robust support for IFIoU’s precise regression, while IFIoU’s targeted regression constraints maximize the localization potential of high-quality features, achieving simultaneous improvements in feature quality and localization accuracy. When integrating ASFF, LSKA, and IFIoU modules concurrently, the model achieves precision, recall, and mAP@0.5 of 93.8%, 93.8%, and 96.9%, respectively. With 18.66 M parameters and 16.85 G FLOPs, with an FPS of 89. All core metrics achieved optimal improvements while maintaining a frame rate significantly exceeding real-time remote sensing detection requirements. This fully demonstrates that the three modules form a comprehensive synergistic enhancement system across three dimensions: cross-scale feature integration, small object feature enhancement, and localization accuracy optimization. Furthermore, they effectively control computational overhead while improving performance, validating the necessity of each improved module’s design and the overall solution’s effectiveness.

**Figure 13:**
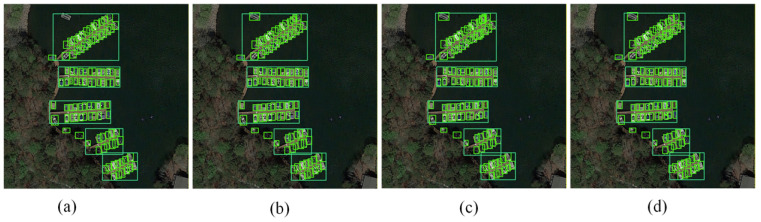
Comparison of small object detection results under different enhancement modules: (**a**) Original YOLOv8s; (**b**) YOLOv8s + ASFF; (**c**) YOLOv8s + ASFF + LSKA; (**d**) AL-YOLOv8 (ASFF + LSKA + IFIoU).

To visually demonstrate the performance gains of each improvement module for detecting small targets in remote sensing, we generated qualitative comparison images through ablation experiments. These sequentially present detection results from the original YOLOv8, YOLOv8 with ASFF, YOLOv8 with LSKA, and the final integrated IFIoU model. As shown in Figure 13: (a) The detection results of the original YOLOv8s exhibit minor missed detections, false positives, and bounding box misalignments in scenarios with dense small objects such as ships in a port. (b) After incorporating the multi-scale feature fusion module ASFF, the model’s ability to represent features of objects at different scales is enhanced, significantly reducing false positive and false negative rates while improving the completeness of detection box coverage. (c) Further introduction of the large-kernel convolutional attention module LSKA improves the model’s fine-grained feature extraction capability for small objects, further optimizing bounding box localization accuracy and achieving higher contour-fitting precision. (d) Finally, integrating the IFIoU loss function optimizes bounding box regression constraints, achieving optimal detection accuracy and consistency with zero false positives or negatives, while significantly reducing box localization errors. This intuitively validates the synergistic effects of ASFF, LSKA, and IFIoU modules in enhancing small object feature representation, optimizing multi-scale fusion, and improving bounding box localization accuracy, fully demonstrating the effectiveness and necessity of each improvement module.

### 4.5. Object Detection Result Comparison

**Figure 14:**
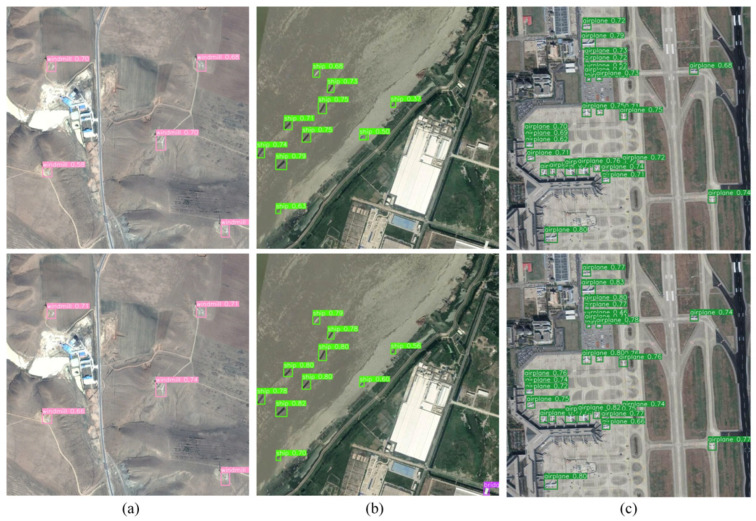
Comparison of Detection Accuracy Before and After Model Improvement (above is YOLOv8s, below is AL-YOLOv8). (**a**) windmill; (**b**) ship; (**c**) airplane.

**Figure 15:**
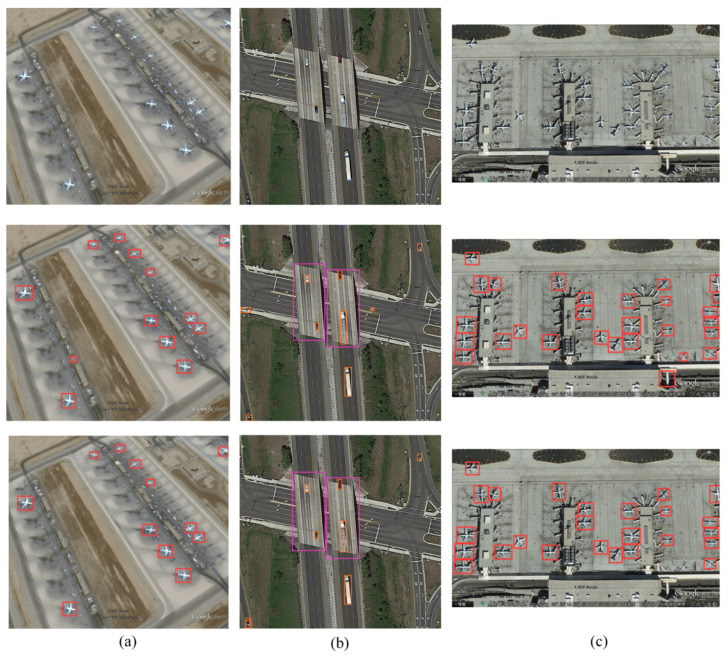
Comparison of False Detection Rates Before and After Model Improvement (top, middle, bottom: original image, YOLOv8s detection results, AL-YOLOv8 detection results). (**a**) airplane; (**b**) vehicle; (**c**) airplane.

To visually validate the improved model’s enhanced accuracy and false alarm resistance in remote sensing small object detection, this section presents performance comparisons through visualizations of detection results across two typical scenarios. The results are shown in Figure 14 and Figure 15.

In Figure 14a, the improvement model exhibits detection confidence levels of 58–70% for windmill targets, with a minor offset in bounding box localization. The improved model not only elevates target detection confidence to 66–74% but also optimizes bounding box alignment with targets, effectively enhancing feature representation for sparse objects.

In Figure 14b, the improvement model exhibits significant fluctuations in detection confidence for ship targets, with some small coastal vessels achieving only 37% and 50% confidence. This reflects the model’s insufficient ability to distinguish small ship features against water ripple backgrounds. The improved model significantly enhances detection confidence for all ship targets, with particularly notable increases of 19% and 10% for small coastal vessels. This enhancement effectively distinguishes ship features from water surface backgrounds, reducing misclassifications.

In Figure 14c, the detection confidence of the improvement model for airplane targets clustered between 62% and 75%, indicating insufficient detection stability in dense small target scenarios. The improved model significantly enhances detection confidence for aircraft targets and achieves more stable detection even in airport edge regions. This result validates the model’s interference resistance in dense small object scenarios. Through attention enhancement and localization optimization, it effectively improves detection stability for densely distributed small targets.

Figure 15 illustrates the false detection comparison between the original and improved models in complex scenarios, visually demonstrating the enhanced false detection resistance of the improved model. In Figure 15a, the scene contains 12 actual aircraft targets. The pre-improvement model detected 13 aircraft, with one false detection caused by the grassy background at the airport edge. The improved model accurately detected all 12 actual aircraft without any additional false positives. In Figure 15b, the scene contains 7 real vehicle targets. The pre-improvement model detected 8 vehicles, with 1 false positive caused by misinterpreting road texture as a vehicle. The improved model accurately detected 7 real vehicles, eliminating false positives caused by background textures. In Figure 15c, the scene contained 25 real aircraft targets. The pre-improvement model detected 27 aircraft, with 2 false positives caused by the edges of airport buildings. The improved model accurately detected all 25 real aircraft, effectively filtering out background interference from building edges. The improved model effectively filtered background interference, retaining only detection boxes for real targets, significantly reducing the false positive rate.

To validate the model’s detection capability for extremely small targets under extreme conditions, targeted experiments were designed on the DIOR dataset. Synthetic raindrops were added to test set images, with raindrop generation parameters based on the China Meteorological Administration’s “Precipitation Intensity Classification Standard”: Intensity factors were uniformly sampled within the range [0.3, 0.8], corresponding to moderate to heavy rain levels; Rain drop density ranges from 900 to 1900 drops/m^2^ (with 1000 ± 100 drops/m^2^ as the moderate rain baseline). Rain drop length is randomly generated between 19 and 34 pixels, corresponding to an optical equivalent physical diameter of 1.9 to 3.4 mm. Simultaneously, the model focuses on ultra-small targets with pixel dimensions below 16 × 16, specifically testing the performance limits for small object detection.

**Figure 16:**
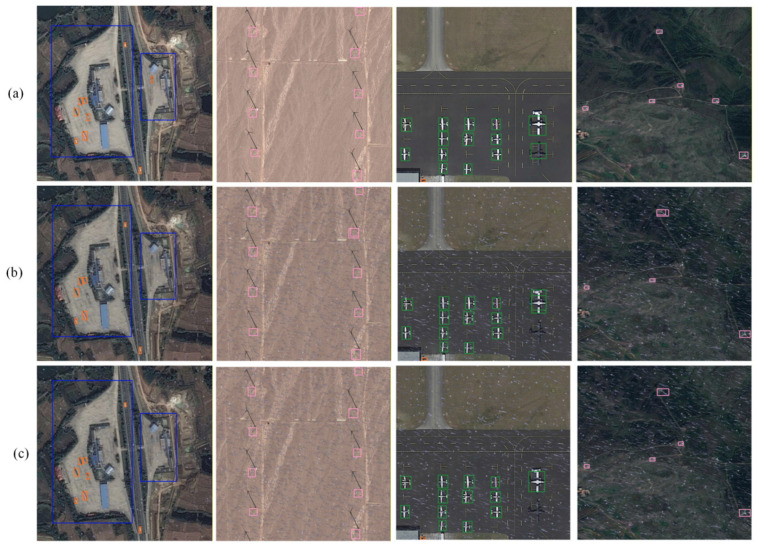
Comparison of Detection Results for Extremely Small Targets in Extreme Conditions. (**a**) Detection results of AL-YOLOv8 on the original test dataset; (**b**) detection results of baseline YOLOv8s under raindrop interference; (**c**) detection results of AL-YOLOv8 under raindrop interference.

The experimental results are shown in Figure 16. Group a shows AL-YOLOv8’s detection results on the original, uninterrupted test set; Group b presents baseline YOLOv8s’ detection results with added raindrop interference; Group c displays AL-YOLOv8’s detection results with added raindrop interference. The results indicate that in Group a, AL-YOLOv8 achieved zero false negatives and false positives for all small targets. In Group b, the baseline model exhibited significant performance degradation under raindrop interference, missing one car, one airplane, and one windmill, while misclassifying one airplane and one windmill. In Group c, the improved AL-YOLOv8 showed only a few missed detections with no false positives.

Failure analysis indicates that even AL-YOLOv8 exhibits minor missed detections under extreme scenarios, suggesting the current architecture still has room for optimization when dealing with interference in harsh environments.

## 5. Discussion and Conclusions

This paper addresses false detection issues in small-target detection from remote sensing imagery caused by complex backgrounds and small object sizes. It proposes the AL-YOLOv8 model, which integrates ASFF adaptive spatial feature fusion, LSKA large-kernel separable attention, and a hybrid IFIoU loss function. Experimental results demonstrate that the improved model outperforms baseline YOLOv8s across three remote sensing datasets—DIOR, RSOD, and NWPU VHR-10—achieving precision rates of 91.5%, 94.2%, and 91.8%, respectively, and mAP@0.5 scores of 89.8%, 96.9%, and 92.2%, while significantly reducing false detection rates. Compared with other mainstream models, our model demonstrates more balanced accuracy and detection stability, particularly excelling in complex scenarios with highly similar backgrounds.

This study has limitations: First, the model was validated only on optical remote sensing datasets and has not been extended to multimodal data such as SAR and hyperspectral data. Second, detection performance for ultra-small targets remains improvable. Future research will focus on two directions: expanding validation datasets to multimodal remote sensing data to enhance model generalization, and designing fine-grained feature enhancement modules for ultra-small targets to improve detection accuracy in extreme scenarios.

AL-YOLOv8 provides an efficient and feasible technical solution for detecting small objects in remote sensing imagery. It can be widely applied in scenarios such as ecological environment monitoring, urban precision management, and territorial spatial planning, providing technical support for the intelligent upgrade of integrated sky–ground monitoring systems. However, during deployment, the following practical constraints must be considered to ensure stable implementation and effective operation.

(a) Image Resolution Requirements for Effective Method Operation

This method is specifically designed for high-resolution optical remote sensing imagery, enabling effective detection of small targets such as aircraft, vehicles, and wind turbines. Its performance relies on the detailed texture and shape information of targets within the imagery. To ensure stable detection accuracy and recall rates, imagery with higher spatial resolution is recommended.

(b) Sensitivity to Atmospheric Distortion

The current model exhibits sensitivity to atmospheric disturbances such as clouds, haze, fog, and light attenuation. Severe atmospheric distortion blurs the boundaries and texture information of small targets, impairing the model’s feature extraction capability. As this method lacks an integrated atmospheric correction module, detection performance may slightly decline under poor atmospheric conditions, limiting its full potential.

(c) Calibration Requirements for Specific Spectral Ranges

Both training and validation of this method were conducted using three-channel RGB optical imagery. Its feature extraction logic and parameter settings are tailored to the spectral characteristics of such imagery. Direct application to other spectral ranges, such as multispectral or hyperspectral remote sensing data, may result in significant performance degradation due to substantial differences in spectral dimensions and radiometric properties, rendering the model unsuitable for practical applications. Therefore, when applying this method to data from other spectral ranges, targeted calibration and fine-tuning are essential to ensure its applicability.

## Figures and Tables

**Table 1:** Number and Proportion (%) of Small Targets in DIOR Dataset.

Category	Total	Small Target Number	Small Target Percentage
vehicle	40,365	40,050	98.97
ship	62,537	60,242	96.33
storage tank	26,403	23,656	89.60
windmill	5363	4584	85.47
airplane	10,100	7482	74.08

**Table 2:** Number and Proportion (%) of Small Targets in RSOD Dataset.

Category	Total	Small Target Number	Small Target Percentage
aircraft	5374	5044	93.86

**Table 3:** Number and Proportion (%) of Small Targets in NWPU VHR-10 Dataset.

Category	Total	Small Target Number	Small Target Percentage
storage tank	655	655	100.00
vehicle	598	594	99.33
ship	302	287	95.03

**Table 4:** Configuration of model training parameters.

Training Parameters	Value
epochs	200
learning rate	0.01
optimizer	SGD
weight_decay	0.0005
warmup_epochs	3.0
warmup_momentum	0.8
Seed	42
batch size	8
image size	640 × 640

**Table 5:** Comparison Results with Other Models.

Model	Precision/%	Recall/%	mAP@0.5/%
FasterR-CNN	87.2	82.6	84.9
CenterNet	87.5	92.1	86.5
Cascade R-CNN	89.0	89.5	91.9
Deformable R-FCN	87.8	88.8	88.3
Sig-NMS	87.5	89.0	90.1
AGMF-Net	91.0	93.5	95.1
YOLOv3	92.3	87.5	95.2
YOLOv5	88.9	91.5	92.4
YOLOv6	89.5	88.3	94.6
YOLOv9	90.3	91.2	92.2
YOLOv10	93.4	92.5	92.8
YOLOv11	90.6	93.8	95.1
AL-YOLOv8	94.2	93.8	96.9

**Table 6:** LSKA Embedding Position Comparison Experiment.

LSKA Position	P/%	R/%	mAP@0.5/%
Before P4 Upsampling	91.6	91.5	95.3
After P3 Feature Layer	91.1	90.7	93.1
After P4 Downsampling	92.1	91.2	94.9
After P5 Feature Layer	93.0	91.9	95.7

**Table 7:** The Impact of Different Ratio Parameters in IFIoU on the Performance of mAP@0.5 (%).

Class	0.1	0.2	0.3	0.4	0.5	0.6	0.7	0.8	0.9
aircraft	92.1	92.7	93.3	93.9	94.5	95.1	95.7	95.1	94.5
all	93.3	93.9	94.5	95.1	95.7	96.3	96.9	96.3	95.7

**Table 8:** Performance Comparison of CIoU and IFIoU on mAP@[0.5:0.95] (%).

Class	CIoU	IFIoU
aircraft	59.9	60.8
all	65.1	66.1

**Table 9:** Comparison of object detection results on the DIOR dataset.

Class	YOLOv8/%				AL-YOLOv8/%			
	P	R	mAP@0.5	mAP@[0.5:0.95]	P	R	mAP@0.5	mAP@[0.5:0.95]
vehicle	87.4	58.9	73.9	45.5	89.2	58.8	76.1	47.8
ship	94.8	90.3	93.9	63.8	95.6	90.4	94.8	65.3
storage tank	94.7	82.9	92.2	64.0	95.3	84.5	92.6	65.4
airplane	98.5	93.3	96.2	76.7	99.2	92.6	97.0	78.6
windmill	95.7	87.0	93.9	53.4	96.4	88.3	94.8	55.3
all	89.7	82.2	88.5	66.8	91.5	83.7	89.8	69.0

**Table 10:** Performance Statistics of the AL-YOLOv8 Model Across Five Independent Experiments on the DIOR Dataset (Mean ± Standard Deviation and 95% Confidence Interval).

Class	Precision P/%		Recall R/%		mAP@0.5/% -		mAP@[0.5:0.95]/%	
	Mean ± SD	95%CI	Mean ± SD	95%CI	Mean ± SD	95%CI	Mean ± SD	95%CI
vehicle	89.2 ± 0.4	[88.7, 89.7]	58.8 ± 0.3	[58.4, 59.2]	76.1 ± 0.4	[75.6, 76.6]	47.8 ± 0.3	[47.4, 48.2]
ship	95.6 ± 0.3	[95.2, 96.0]	90.4 ± 0.4	[89.9, 90.9]	94.8 ± 0.3	[94.4, 95.2]	65.3 ± 0.4	[64.8, 65.8]
storage tank	95.3 ± 0.4	[94.8, 95.8]	84.5 ± 0.5	[83.9, 85.1]	92.6 ± 0.4	[92.1, 93.1]	65.4 ± 0.5	[64.8, 66.0]
airplane	99.2 ± 0.2	[99.0, 99.4]	92.6 ± 0.4	[92.1, 93.1]	97.0 ± 0.3	[96.6, 97.4]	78.6 ± 0.4	[78.1, 79.1]
windmill	96.4 ± 0.5	[95.8, 97.0]	88.3 ± 0.6	[87.6, 89.0]	94.8 ± 0.5	[94.2, 95.4]	55.3 ± 0.6	[54.6, 56.0]
all	91.5 ± 0.4	[91.0, 92.0]	83.8 ± 0.5	[83.2, 84.4]	89.8 ± 0.4	[89.3, 90.3]	69.0 ± 0.4	[68.5, 69.5]

**Table 11:** Comparison of False Detection Types in Complex Scenarios Using the DIOR Dataset and False Detection Suppression Effectiveness of AL-YOLOv8.

False Alarm Type	YOLOv8s	AL-YOLOv8	Reduction Rate/%
Road texture → vehicle	35	9	74.29
Water surface → ship	12	2	83.33
Industrial ground → storage tank	18	4	77.78
Grass background → airplane	28	5	82.14
Airport building edge → airplane	19	4	78.95
Farmland → windmill	8	1	87.50

**Table 12:** Comparison of object detection results on the RSOD dataset.

Class	YOLOv8/%				AL-YOLOv8/%			
	P	R	mAP@0.5	mAP@[0.5:0.95]	P	R	mAP@0.5	mAP@[0.5:0.95]
aircraft	94.4	85.4	92.4	59.9	95.7	86.2	95.7	60.8
all	91.6	91.9	95.8	65.1	94.2	93.8	96.9	66.1

**Table 13:** Comparison of object detection results on the NWPU VHR-10 dataset.

Class	YOLOv8/%				AL-YOLOv8/%			
	P	R	mAP@0.5	mAP@[0.5:0.95]	P	R	mAP@0.5	mAP@[0.5:0.95]
airplane	98.2	99.4	98.9	67.8	98.9	99.8	99.5	71.0
vehicle	79.1	78.0	78.8	52.6	82.5	78.6	90.5	67.3
storage tank	96.6	98.6	89.5	65.1	97.4	99.5	99.0	65.8
ship	77.6	81.6	79.0	52.5	81.2	81.4	83.6	57.3
all	89.8	87.7	90.1	58.0	91.8	88.9	92.2	62.8

**Table 14:** Ablation Experiment Results.

ASFF	LSKA	IFIoU	P/%	R/%	mAP@0.5/%	Parameters/M	FLOPs	FPS
—	—	—	91.6	91.3	95.1	11.14	14.33	114.1
√	—	—	93.5	92.5	95.6	16.58	18.55	87.3
—	√		93.0	91.9	95.7	11.41	14.43	115.7
—	—	√	93.1	92.7	95.8	11.14	14.33	115.6
√	√	—	93.3	93.3	95.9	18.66	16.85	90.8
√	—	√	93.7	93.0	96.2	16.58	18.55	86
√	√	√	94.2	93.8	96.9	18.66	16.85	89

## Data Availability

The public dataset used in this paper is available at https://aistudio.baidu.com/datasetoverview (accessed on 9 March 2024).

## Abbreviations

The following abbreviations are used in this manuscript:

ASFF	adaptive spatial feature fusion
LSKA	Large-kernel separable attention
C2f	Cross Stage Partial with 2 Fusion
SPPF	Spatial Pyramid Pooling-Fast
PAN	Path Aggregation Network
FPN	Feature Pyramid Network
LGDD	Lightweight GroupNorm Detail-enhance Detection
SPD	space-to-depth
HiDRA	Hierarchical Dynamic Refinement Attention
DCDBlock	Densely Connected Dilated Block
DCBANet	Dynamic Context Branch Attention Network
DCSA	Dynamic Context Scale-Aware
CASM	Context-Adaptive Selection Module
